# Complete Genome Sequence of *Aurantimicrobium minutum* Type Strain KNC^T^, a Planktonic Ultramicrobacterium Isolated from River Water

**DOI:** 10.1128/genomeA.00616-16

**Published:** 2016-06-30

**Authors:** Ryosuke Nakai, Takatomo Fujisawa, Yasukazu Nakamura, Hiroyo Nishide, Ikuo Uchiyama, Tomoya Baba, Atsushi Toyoda, Asao Fujiyama, Takeshi Naganuma, Hironori Niki

**Affiliations:** a Genetic Strains Research Center, National Institute of Genetics, Mishima, Japan; b Superlative Postdoctoral Research Fellow of the Japan Society for the Promotion of Science, Tokyo, Japan; c Center for Information Biology, National Institute of Genetics, Mishima, Japan; d The Graduate University for Advanced Studies (SOKENDAI), Mishima, Japan; e Data Integration and Analysis Facility, National Institute for Basic Biology, National Institutes of Natural Sciences, Okazaki, Japan; f Laboratory of Genome Informatics, National Institute for Basic Biology, National Institutes of Natural Sciences, Okazaki, Japan; g Transdisciplinary Research Integration Center, Research Organization of Information and Systems, Tokyo, Japan; h Principles of Informatics Research Division, National Institute of Informatics, Tokyo, Japan; i Graduate School of Biosphere Science, Hiroshima University, Higashi-Hiroshima, Japan

## Abstract

*Aurantimicrobium minutum* type strain KNC^T^ is a planktonic ultramicrobacterium isolated from river water in western Japan. Strain KNC^T^ has an extremely small, streamlined genome of 1,622,386 bp comprising 1,575 protein-coding sequences. The genome annotation suggests that strain KNC^T^ has an actinorhodopsin-based photometabolism.

## GENOME ANNOUNCEMENT

The phylum *Actinobacteria* includes members that are often numerically dominant (up to 60%) in the freshwater bacterial communities (1, 2). The actinobacterial Luna2 lineage, currently classified into the acIII lineage (2), has a cosmopolitan distribution in inland freshwater ecosystems (3). The cell morphology of this lineage is typical of ultramicrobacteria (cell volume <0.1 µm^3^ [4]). *Aurantimicrobium minutum* type strain KNC^T^ was the first described strain with a validly published name within the acIII lineage of *Actinobacteria* (5) and was isolated from a 0.2 µm filtrate of river water in western Japan (6). Here, we report the whole-genome sequence of strain KNC^T^, with the aim of elucidating the physiological traits that have facilitated such a wide distribution pattern.

The genomic DNA of strain KNC^T^ was extracted from cells grown in nutrient broth, soytone, yeast extract (NSY) liquid medium (7) using Qiagen Genomic-Tip 100/G columns. Genomic shotgun and fosmid-end sequences were determined using an ABI3730xl sequencer. *De novo* assembly was conducted with phrap version 1.080812, resulting in a single chromosome with 22.2-fold genome coverage. The total length of the complete genome was 1,622,386 bp with a G+C content of 52.8 mol%. Strain KNC^T^ has an extremely small, streamlined genome that is consistent with other ultramicrobacteria such as “*Candidatus* Pelagibacter ubique” (8) (*Alphaproteobacteria*) and *Rhodoluna lacicola* (9) (*Actinobacteria*). The phylum *Actinobacteria* is generally considered to be comprised of high-G+C Gram-positive bacteria. However, the genomes of freshwater and marine actinobacteria were recently reported to have unusually low G+C contents (9, 10). The genomic G+C content of freshwater strain KNC^T^ is also quite low.

The genome annotation by the Microbial Genome Annotation Pipeline online server (11) predicted one copy of 16S-23S-5S rRNA operon, 42 tRNAs, and 1,575 protein-coding sequences. The genome encoded a putative rhodopsin, known as actinorhodopsin (12), suggesting that strain KNC^T^ has an ability to utilize light energy for supplemental energy generation. Conversely, genes encoding a cytochrome *bd* complex, a respiratory quinol:O_2_ oxidoreductase found in many prokaryotes and expressed under oxygen-limited conditions (13), were missing within the genome. Comparative genomic analysis by MBGD (14) revealed that the genomic traits described above are shared with the genome of another ultramicrosized actinobacterium *R. lacicola* (9). In summary, we sequenced the complete genome of a cosmopolitan freshwater ultramicrobacterium. This will provide new insight into genome streamlining and related missing genes of ultramicrobacteria.

### Nucleotide sequence accession number.

The complete genome sequence of *A. minutum* KNC^T^ has been deposited in the DDBJ/ENA/GenBank database under the accession no. AP017457. Annotated genome data were deposited with the help of the Genome Refine web service (http://genome.annotation.jp/genomerefine/).
